# Alveolar Abscess

**Published:** 1863-07

**Authors:** Wm. H. Atkinson

**Affiliations:** New York


### Selections.

---

ALVEOLAR ABSCESS.—*By Wm. H. Atkinson, M. D.—Read before the "Brooklyn Dental Association."*—Alveolar Abscesses, like abscesses in general, may be divided primarily into cold and warm, slow or chronic, and rapid or acute. The former contain broken tissue, heterogeneous and poorly defined; the latter are filled with exudates of histogenic elements in various degrees of progress, toward organization of which pus composes the principal portion; in a word, a proteinaceous compound, resultant upon the solution of the tissues, which have suffered disintegration by the action of local disease.

Cold abscesses may be recognized by enlargement of the part, a clammy, doughy sort of feel, like œdema in dropsical habits of body; by the absence of considerable tenderness or constitutional disturbances. These must be evacuated, and dressed with stimulating remedies, at the same time supporting the general system by proper tonic and detergent treatment.

There are many degrees of mergence between the cold and warm varieties of abscess, and of course some will be exactly midway between the extremes, rendering the diagnosis difficult until after opening, when the contents will at once inform us of the specific character of the example under examination.

The warm variety is attended by tenderness, swelling, and redness, to a greater or less extent, whose tumor softens in from twenty-four hours to eight days, presenting the phenomenon of fluctuation to the educated sense of touch—which is the true indication to promptly evacuate—when we will find well-digested pus and blood are the only discharges coming from the cut—the pus coming from the centre, and the fresh blood and fibrinous exudate from near the walls of the tumor.

In these cases, simple dressings or tents are all that is required to insure rapid recovery, the constitutional vigor having already discharged the germs of local disease into the abscess, thereby relieving the system of the cause of systemic disturbance, against the recurrence of which we are secured so soon as this is completely drained of its contents.

These last occur in good constitutions, and have rapid course and sound recovery; while the former, viz: the cold, occur in poorly endowed systems, of scrofulous or other cachectic habit, are slow in progress and tedious of cure, even under intelligent and well-directed treatment, faithfully obeyed on the part of the patient. All these last demand more than local treatment and care.

The rule for local treatment of all varieties of abscess is to apply only such topical remedies as tend to the comfort of the patient at the time, whether they be hot, cold, or lukewarm.

The active or inflammatory cases usually are most comforted by cold; the cold, slow, and phlegmatic by warm; and the mixed by alternations of hot, cold, and lukewarm dressings in such quick succession as to produce some shock to the neural and vascular circulations inducing fuller and freer afflux of the life-flow to the part.

Wherever there is malignity of any considerable degree present, detergents must be used locally and generally; but where only debility and benign lesion hold dominion, milder dressings are called for and tonic remedies internally administered.

The saturated solution of resublimed iodine in pure creosote is the best known remedy for malignant abscesses, but "vin. opii" is preferable as a local dressing for the benign from the first, or after the malignity has been antidoted by the proper treatment. So soon as the germs of disease are expelled systematically and locally, we have to deal only with simple lesion of continuity of the structures involved. A failure to apprehend this fact and recognize its presence has delayed the closure of many such lesions.

Over-treatment, constitutionally or locally, is nearly as much to be deprecated as under or adverse administrations. The fact is, there is no certainty outside of the recognition of, and obedience to, general and local laws. Until the eye becomes conversant with the expressions of health, it can have no knowledge of disease. So various is the standard of even healthy appearances in diverse constitutions, that no single character can establish and determine the status of the part under inspection.

What has been denominated healthy granulations it is hard to define in words alone; but when clinical cases are at hand, the difficulty of teaching the pupil how to designate healthy and diseased states is removed, and teaching made tolerably easy.

The plasm, exudate, or gelatinous plastic mass, which nature weeps out of benign lesions for the purpose of closing them up and forming new tissues, differs so much in various temperaments and conditions of systemic vigor, that it requires the expert to pronounce with certainty whether it be healthy or not, and whether it should be removed as effete or retained as truly proteinaceous, and indispensable to the restoration of the part. Hence clinical, conversational instructions are infinitely more valuable to the learner than the most erudite and clearly expressed writings alone. Two senses, brought to bear, seal the instruction and make the knowledge the pupil's own property.

I have said cure of abscess was secured by evacuation and support. Evacuate the germs of disease and the effete substances, whatever they may be, and support the system up to the point to bear the loss of the blood and proteinaceous plasm that always escapes, more or less, with the effete matters, whether they be soft or hard.

A principal means of securing that necessary support is to be careful to form a pocket to retain the plasm "in situ," so that the metamorphosis of its elements can express itself by progressively forming the histogenic elements into the proper stages of vitalization, organization, and correlation of parts to form nerve, vessel, or bone; one or all which will be arrested or prevented if the pocket is pervious, allowing the plasm to escape and be lost.

Many cases of spontaneous cure of both unruptured and ruptured abscesses are the result of *mechanical support* of the lips and well-organized adjacent soft parts, whose compression, by voluntary or involuntary effort and resilience of structure, cuts off the free supply of blood to the part, and thus induces contraction and condensation of the sac and absorption of its contents, without exciting constitutional disturbance to such degree as to call especial attention of patient or practitioner.

The shrewd observer will take the hint, and imitate nature by the use of well-adapted compresses to all cases that are of such character as to bear them from the first; and such as are not, until after some progress toward ripening or cure has been effected, will be very much hastened to a favorable termination by a judicious use of these opportune appliances.

The mechanical support afforded to the debilitated parts by *poultices* is their chief recommendation, all else being concomitant with, or adventitious to, this prime prerequisite of local treatment. Heat, cold, stimulating, sedative, narcotic, and electroid-adjuvant applications, all have their indications and uses, which, when intelligently selected and alternated, marvelously comfort the patient and facilitate rapidity and permanency of cure.

There is a stage of the progress of the formation of abscess in which it is injudicious to use the knife. I refer to the stage of incubation or state of mergence from a congested, solidified condition to a fluid one, which has two indications—the one favorable, and tending to solution of the tissues in healthy degree; and the other unfavorable, and productive of necrosis of blood, exudate, or bone, which last are enhanced by mechanical violence, if applied during the state of change. It is also unfortunate for the recovery of the parts if we evacuate this solution when benign; but, after necrosis has taken place, it is then the best possible practice to remove the effete matters at once, thus limiting the size and virulence of the abscess.

In case of necrosis of the alveolar plates, from syphilis or other cachexia, extraction of the teeth is strongly contra-indicated. Constitutional treatment, conjoined with local enucleation of the periosteum from the necrosed or necrosing bone, and its prompt removal, so soon as the line of demarkation has been well defined, saving the teeth and gums intact, as far as it is possible to do so, and get away the dead sections of the plates or processes, is the only legitimate and scientific treatment for such cases. Where this is carefully and faithfully done, the teeth are preserved in perfection and new sockets are formed, giving them even firmer attachment than the original formations afforded in the majority of instances.

Thus we preserve the natural articulation of the teeth and contour of the jaws and regularity of the arch of the roof of the mouth, defying the sharpest scrutiny to detect that the lesion had ever been present. But where the enucleation has been imperfectly effected, the periosteum torn or folded, irregularities of the new formations indicate to the competent observer that disease had once held dominion there.

Surgeons and dentists have usually, I might say universally, esteemed the presence of the teeth obstacles to the proper treatment and recovery of the case, which is simply a great mistake.

The attachment of the free borders of the gums to the teeth affords us the requisite pocket to hold the plasm or exudate, out of which to construct the new parts; and the fangs of the teeth are like the scaffold poles of a building, around which to build the plastic materials which construct the parts, in so much less time and with greater facility and perfection than in cases where they are missing.

Conservative surgery here, as elsewhere, is beginning to assert its rights and prove its supremacy over the wholesale amputations and destruction of parts, so common among the reigning dynasty of surgeons and dentists.

Reproductions of bones, tendons, blood-vessels, and nerves is now too common to be seriously questioned by the intelligent observer; but that of muscle, gland, and epidermis has not yet become of familiar occurrence among mammals, where these have been lost by solution or traumatic removal. Because we have not seen these, it does not entitle us to assert that it never can be accomplished. Ten years ago not a single Amen could be evoked in response to my efforts in the direction of curing and the doctrine of curability of alveolar abscess. Until that time, the effort had been a lone struggle against difficulties, timidly but perseveringly followed. But now there are various parties who make light of it, and claim to be "able to accomplish sound cures in a very few days of the worst cases," who have not even the ability to diagnose and distinguish abscess from tumor, nor simple phlegmon of the gums (gum-boil) from serious forms of true "alveolar abscess!" This is not astonishing, for it is the prerogative "par excellence" of ignorance to assume to be wanting in nothing; while earnest desire to be first in usefulness, is modest in pretension, and persevering in effort, and open to convictions of truth, from whatever origin. I should not be surprised to hear, in a few years or months, that reproductions of alveolar processes and the bodies of the maxillary bones was nothing new, but, like the mallet, a mode "long since tried, and laid aside for want of utility and safety." But by whatever and by all means, "so that the truth shall be preached," and *prevail*, through "malice, envy, or good-will," the progressive mind "does rejoice, and will rejoice!"

To sum up, then. Alveolar abscess does exist and prevail to an alarming extent; and if better practices do not soon take the field, it is destined to become a scorching opprobrium to the science of the dental profession. And if natural organs are preferable to miserable substitutes, it calls loudly on all in this field of practice to qualify themselves to fulfill the demands arising from the needs of the community in this respect, or they will be superseded by "branches cut from the olive tree that is wild by nature," that will fulfill this necessity!

Cure the benign forms by local treatment, and malignant by constitutional and local.

Local consists in evacuating; constitutional, in antidoting the cachexia by administration of the forms of the preparations of iodine, mercury, and potash, pro re nata.

*Polychrest Remedies.*—For local dressing, creosote alone, and with iodine dissolved in it, and tinctures of iodine in alcohol and glycerin, wine of opium and solution of the sulphate of alumina and potash.

For internal administration, iodine of mercury and opium, drages of hydriodate of potash, separate or combined, alternately or simultaneously.

New York, 1863.

